# A Tuneable and Easy-to-Prepare SERS Substrate Based on Ag Nanorods: A Versatile Tool for Solution and Dry-State Analyses

**DOI:** 10.3390/nano14221808

**Published:** 2024-11-11

**Authors:** Margherita Longoni, Sofia Zucca, Silvia Bruni

**Affiliations:** Dipartimento di Chimica, Università degli Studi di Milano, Via Golgi 19, 20133 Milan, Italy; margherita.longoni@unimi.it (M.L.); sofia.zucca@studenti.unimi.it (S.Z.)

**Keywords:** SERS spectroscopy, silver nanorods, tuneable substrate, drystate, SSE™ Raman, historical natural dyes

## Abstract

Surface-enhanced Raman spectroscopy is a powerful technique for the ultra-sensitive detection of organic analytes. In this paper, the preparation of SERS substrates based on silver nanorods (AgNRs) is proposed, exploiting a simple protocol which does not require complex procedures and/or sophisticated and expensive instrumentation. For this purpose, various syntheses of AgNRs were tested, and the best one for preparing the SERS active substrate proved to be the one which does not involve surfactants as nanoparticle stabilizers. The plasmonic properties of the selected substrate can be modified based on the concentration of the deposited nanoparticles, allowing for the experimentation of different excitation wavelengths. Positive results were obtained on reference solutions of three natural dyes of historical interest using both green exciting radiation (532 nm) and two near-infrared ones (785 and 850 nm; the latter is combined with the SSE™ technology for further fluorescence quenching). Furthermore, the substrates of AgNRs were found to be suitable for SERS measurements even in dry-state conditions, i.e., only exploiting the electromagnetic interaction between the nanostructured substrate and the dye molecules absorbed onto a wool fibre.

## 1. Introduction

Surface-enhanced Raman spectroscopy (SERS) is a well-known vibrational spectroscopic technique for the detection of organic compounds, even at the single-molecule level, in a wide range of application fields, from biomedicine to food science and cultural heritage diagnostics [1,2]. Indeed, SERS allows for the structural fingerprinting of low-concentration organic analytes, overcoming the limitations associated with traditional Raman spectroscopy (the weakness of the signals and the fluorescence background) but retaining at the same time its advantages and, in particular, its specificity in molecular recognition.

In this respect, a key role in SERS is played by the interaction between the analyte molecules and a nanostructured metal substrate. The latter, in fact, is responsible for the amplification of the Raman signals of up to a factor of 10^8^ and, meanwhile, allows the quenching of most of the fluorescence photons emitted by the organic substances [3]. The most typical SERS substrates are colloidal suspensions of gold, silver, and copper nanoparticles, as they can be quickly prepared and easily used. However, in recent decades, the preparation of solid substrates, i.e., self-assembly of nanoparticles built on a rigid support, more stable and easier to use, has been proposed, but often their synthesis is very complex and time-consuming and also requires the use of specialized equipment [4].

Recently, a simple protocol was proposed to produce films obtained from drop depositions of silver colloids on an optically transparent support, such as a functionalized glass slide, aiming at preparing an accessible and easy-to-produce SERS substrate, which does not require complex procedures and sophisticated instrumentation and is suitable for in situ analysis of cultural heritage objects by means of portable Raman instrumentation [5]. In particular, it was demonstrated that anisotropic nanoparticles (such as silver nanostars [6]), in addition to giving rise to highly concentrated local electromagnetic fields, lead to further amplification of the Raman scattering in SERS [7,8] and are able to interact directly to form hot spots (areas of strong SERS intensification of the Raman signal formed between close nanoparticles) without requiring activation by aggregation. This allows us to avoid inhomogeneity of the surface due to the addition of an electrolyte as an aggregating agent.

In this work, silver nanorods (AgNRs) were exploited as anisotropic nanomaterials to prepare SERS-active substrates, following the same simple protocol developed in [5], which involves the functionalization of a glass slide with (3-aminopropyl)trimethoxysilane (APTMS) followed by the drop-deposition and slow drying of a small volume of preconcentrated nanoparticles. Silver nanorods have already been exploited for SERS applications, especially in the form of solid arrays, although the required procedures are often extremely complex [9,10,11,12,13]. Herein, we experimented with the preparation of SERS-active substrates starting from three different AgNRs, two specifically designed for the application of SERS (Rheka [9] and Volkan [10] AgNR) and one not yet exploited with this technique (Mahmoud [14] AgNR). Obtaining Ag nanorods requires the use of capping agents to promote the growth of the nanoparticles along a single direction [15], which for the first two colloids was a surfactant and a polymer for the third one. The three colloids were characterized by UV-visible spectroscopy and deposited to obtain the solid substrates, which were studied via scanning electron microscopy (SEM) with an energy-dispersive X-ray (EDX) microprobe.

Thus, the substrates obtained were then exploited for SERS measurements on reference solutions of natural dyes of historical interest (alizarin, purpurin, and lac dye) using different excitation wavelengths (532, 785, and 850 nm). It was, in fact, demonstrated that by varying the concentration of the deposited nanoparticles, it is possible to tune the plasmonic properties of the final substrate. In particular, the use of a handheld Raman spectrometer exploiting a near-infrared (NIR) excitation at 850 nm and based on SSE™ (Sequentially Shifted Excitation) technology, was tested for the first time in combination with this kind of substrates, as both conditions allow for further quenching of the fluorescence signal. The synthesis of the SERS sensors compatible with this instrument may in fact be of great interest, since it has the notable advantage of compactness and transportability, which make it very suitable for in situ analyses in different application fields. Finally, the AgNR substrates were also tested in the dry state, i.e., to analyze alizarin molecules absorbed onto a dyed wool fibre. In this condition, the interaction between the nanostructured substrate and the analyte is more challenging, since only the electromagnetic component of the SERS effect can be exploited. Nonetheless, as already demonstrated in [5], this is possible, although, it requires a SERS substrate with certain properties (a combination of transparency to allow the passage of the laser, Raman scattering, and the aggregation state that ensures the presence of hot spots). This kind of application is of great interest especially in the field of cultural heritage diagnostic, where a non-invasive approach is strictly recommended to promote the conservation, valorisation, and restoration of artworks.

## 2. Materials and Methods

### 2.1. Materials

Silver nitrate (AgNO_3_, 99.9999%), 3-(aminopropyl)trimethoxysilane (APTMS, 97%), cetyltrimethylammonium bromide (CTAB), sodium borohydride, ascorbic acid, ethylene glycol (EG, ≥99%), polyvinylpyrrolidone (PVP, MW 40,000), sodium hydroxide (NaOH), sulfuric acid (95% to 97%), methanol (≥99.9%), alizarin, and purpurin were purchased from Merck Life Science (Milano, Italy); lac dye was purchased from Zecchi (Firenze, Italy).

### 2.2. Synthesis of Silver Nanorods

#### 2.2.1. Rekha AgNR

Silver nanorods were synthesized using the seed-mediated method described in [9]. To prepare the seeds, 100 mL of a 0.01 M AgNO_3_ solution was combined with 400 µL of a 0.1 M cetyltrimethylammonium bromide (CTAB) solution and 10 mL of a 0.01 M NaBH_4_ aqueous solution. The mixture was stirred for two minutes and then allowed to stand for one hour. For the growth solution, 50 mL of a 0.01 M CTAB aqueous solution was prepared under magnetic stirring and gentle heating to facilitate the dissolution of the surfactant. Subsequently, 1.25 mL of a 0.02 M AgNO_3_ solution and 2.5 mL of 0.1 M ascorbic acid (the reducing agent) were added, resulting in a yellow colour. Finally, to obtain the nanorods, a volume of seed solution (from 625 to 375 µL) was injected into the growth solution along with 500 µL of 1 M NaOH to adjust the pH, promoting growth and nanorod formation. After ten minutes, a dark-coloured nanorod colloid was obtained. The final length of the nanorods depends on the volume of seeds added to the growth solution: a greater amount of seeds leads to shorter rods. Specifically, nanorods were synthesized by adding 625, 400, and 375 µL of seeds, corresponding to aspect ratios of approximately 6–7, 11–12, and 15–18, respectively [9]. Their UV-visible spectra, shown in Figure S1a (Supplementary Materials), are dominated by two plasmonic bands corresponding to the transverse surface plasmon resonance (TSPR) and longitudinal surface plasmon resonance (LSPR). The TSPR is centred around 405–415 nm, while the LSPR shifts from 500 to 600 nm depending on the seed volume added to the growth solution. The final silver concentration was calculated to be approximately 10^−4^ M.

#### 2.2.2. Volkan AgNR

For the synthesis, the method outlined in [10] was used. First, the seeds were prepared: 40 mL of a solution containing 0.25 mM AgNO_3_ and 0.25 mM trisodium citrate were stirred for 2 h with 1.2 mL of a cold 0.010 M NaBH_4_ aqueous. Next, the growth solution was prepared by mixing 7.0 mL of a 0.020 M CTAB solution, 0.50 mL of a 10 mM AgNO_3_ solution, 0.50 mL of a 0.10 M ascorbic acid solution, and 0.50 mL of the seed solution. To adjust the pH to around 11, 0.5 mL of a 1.0 M NaOH solution was added, and the resulting growth solution was allowed to sit for 2 h. The UV-visible spectrum of the resulting nanorod colloid (shown in Figure S1b, Supplementary Materials) displays an absorption band at 415 nm, along with a shoulder at approximately 530 nm, corresponding to the TSPR and LSPR modes, respectively. The average length of these silver nanorods is 70–80 nm, with aspect ratios of about 4 to 5 [10]. The final concentration of silver in the nanorod colloid was estimated to be approximately 5 × 10^−4^ M.

#### 2.2.3. Mahmoud AgNR

These nanorods were synthesized according to the procedure reported in [14]. Briefly, 70 mL of ethylene glycol (EG) were left to reflux at 140 °C for 1 h under constant magnetic stirring (300 rpm). The temperature was then raised to 175 °C and 0.5 g of polyvinylpyrrolidone (PVP) were added, increasing the stirring rate to 1300 rpm. A solution with 0.15 g of AgNO_3_ in 3 mL of EG was then added, and the mixture was left under stirring until the colour transition from orange to dark green was observed (approx. 3 min). The solution was transferred to a beaker in an ice bath and was stirred at 1300 rpm for another 5 min, after which the colloid was ready. EG and byproducts were separated from the silver nanorods by adding 3 mL of ultrapure deionised water to 5 mL of a nanorod solution and via centrifuging at 6000 rpm for 5 min. The precipitate was dispersed in DI water, centrifuged twice again, and finally redispersed in 5 mL of ultrapure deionised water. The colloid was stored in the refrigerator and subjected to sonication before use.

The effectiveness of the nanorod synthesis was controlled by UV-visible spectroscopy: two bands at 435 and 970 nm dominate the absorption spectra modes (Figure S1c, Supplementary Materials), due, respectively, to the transversal (high energy) and longitudinal (low energy) electronic oscillation, as reported in [14]. The average length of these silver nanorods is around 100 nm and their aspect ratios are about 5 [14]. The Ag concentration in the nanorod colloid was approximatively 10^−2^ M.

### 2.3. SERS Substrate Preparation

#### 2.3.1. Functionalization of Glass Support

The protocol described in [5] was followed. In summary, a microscope glass slide was submerged in a 1:1 mixture of methanol and HCl for 30 min, then thoroughly rinsed with distilled water until a neutral pH was achieved, before it was dried under a nitrogen stream. The slide was then dipped in concentrated H_2_SO_4_ for another 30 min, after which the washing and drying steps were repeated as before (washing phase). Next, the glass slide was soaked for 15 min in a solution composed of 45 mL methanol, 2.5 mL ultrapure deionized water, and 2.5 mL (3-aminopropyl)trimethoxysilane (APTMS). It was then carefully rinsed by dipping it in methanol followed by water to remove any remaining APTMS, and finally heated in an oven at 90 °C for 30 min (immobilization phase). The so-prepared slides should be used on the same day.

#### 2.3.2. Drop Deposition of AgNR Colloids

The AgNR colloids were centrifuged, with the time and speed for optimal deposition depending on the colloid type, typically ranging from 5 min at 6000 rpm to 20 min at 5000 rpm. After centrifugation, the colloids were re-dispersed in water to reach the required concentration factor (between 1:50 and 1:100). To achieve this, the supernatant was discarded, and an appropriate volume of water was added to the nanoparticle pellet collected at the bottom of the centrifuge tube. The suspension was then thoroughly mixed by rapid magnetic stirring. A micro-drop (10 µL) of the prepared colloid was then placed on the functionalized glass slide using a micropipette and left to dry in an oven at 50 °C for approximately 2 h, inducing slow evaporation of the solvent to achieve a homogeneous film.

### 2.4. SERS Analyses Using AgNR Substrates

For the SERS analysis of the solutions, a micro-drop (3 µL) was deposited onto the nanostructured surface of the substrate, and the laser was focused on this area. All dye solutions were prepared in methanol at a concentration of 10^−4^ M.

**Figure 1:**
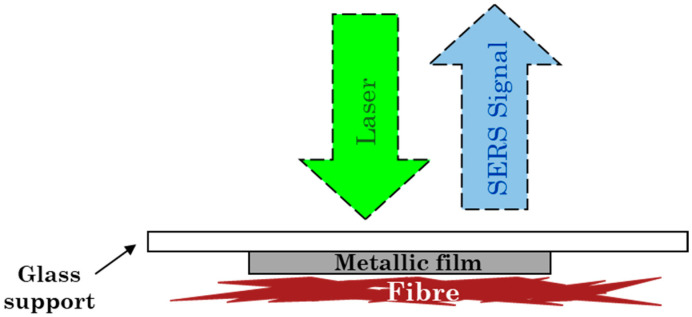
Schematic setup for dry-state SERS analysis on dyed wool thread samples.

The setup for the dry-state analysis is illustrated in Figure 1. A wool thread, dyed with alizarin, was placed in close contact with the SERS substrate, and measurements were taken by focusing the laser beam through the AgNR substrate. In this area, both a nanoparticle aggregate and the dyed wool thread beneath were recognizable through the SERS substrate, thanks to the camera integrated with the Raman instrument.

### 2.5. Instrumentation

#### 2.5.1. UV-Visible Spectroscopy

UV-visible spectra were recorded in the 400–1000 nm spectral range using a Jasco UV/VIS/NIR V-570 spectrometer (Jasco Europe, Cremella, LC, Italy), equipped with both a photomultiplier and a PbS detector. The instrument enables measurements in transmission mode and, when fitted with an integrating sphere, in reflection mode.

#### 2.5.2. Scanning Electron Microscopy Coupled with Energy Dispersive X-Ray Analysis (SEM-EDX)

SEM images were acquired with a Hitachi TM 1000 microscope (Nanovision, Brugherio, MB, Italy), with a resolution of 1 nm and integrated with an energy-dispersive X-ray (EDX) spectrometer. The accelerating voltage was set at 15 kV.

#### 2.5.3. Portable Raman Microprobe

For the micro-SERS analyses, a Jasco RMP100 portable Raman micro-probe (Jasco Europe, Cremella, LC, Italy) was used and equipped with a notch filter to block Rayleigh scattering and a 50× objective. The probe was connected via optical fibres to the laser source and a Lot Oriel MS125 spectrometer, which includes an Andor CCD detector cooled by a Peltier device (1028 × 256 pixels, 26 μm/pixel, operating at −60 °C). Two laser sources were used: a frequency-doubled Nd laser at 532 nm and a diode laser at 785 nm. A grating with 1800 lines/mm (8 cm^−1^ resolution) was used for the first wavelength, and a 1200 lines/mm grating (3 cm^−1^ resolution) for the second. The incident power was always restricted to a few milliwatts. The spectra were recorded in the range 2000–200 cm^−1^, as the sum of 30 scans with a 2 s exposure per scan.

#### 2.5.4. SSE™ Raman Spectrometer

A Bruker BRAVO handheld spectrometer (Bruker Italia, Milano, Italy) was also used for the SERS analyses. This device employs the patented SSE™ technology, which excites spectra using two diode lasers operating at different temperatures, emitting at 785 nm and 850 nm, respectively. An integrated algorithm processes the final Raman spectral data. Spectra are acquired in two sequential steps, covering the ranges from 300 cm^−1^ to 2000 cm^−1^ and from 2000 cm^−1^ to 3200 cm^−1^. The average spectral resolution is around 11 cm^−1^, and the laser power used is always below 100 mW for both lasers. The acquisition time ranges from 500 ms to 2 s, with the number of accumulations varying between 5 and 300, as automatically adjusted by the instrument.

## 3. Results and Discussion

As can be appreciated from the SEM images shown in Figure S2 (Supplementary Materials), the appearance of the substrates obtained via the deposition of the Rehka and Volkan Ag nanorods was not characterized in any of the cases by a consistent coverage of nanoparticle aggregates, a condition associated with a satisfactory SERS response as shown in [5]. One reason for this can be found in the low Ag concentration of the starting colloids (of the order of 10^−4^ M). Furthermore, in both cases, appreciable residues of the CTAB surfactant, recognizable from the bromine peak in the EDX spectrum and also by a matt and glossy appearance of the film or by the presence of scale-shaped structures, could be observed, surrounding or covering the metal nanoparticles (Figure S2b,d, Supplementary Materials). These two facts can explain why, even if both substrates gave a SERS signal for alizarin in the methanolic solution with an excitation at 532 nm (Figure S3, Supplementary Materials), neither allowed for the use of a NIR excitation wavelength or has proven suitable for dry-state measurements.

**Figure 2:**
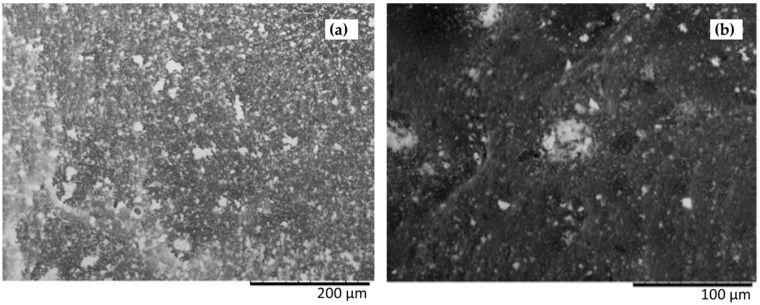
SEM images of Mahmoud AgNR substrates with Ag concentration factor (**a**) 1:50 and (**b**) 1:5.

The choice of a synthesis of the Ag nanorods that does not involve the use of a surfactant but instead of PVP as a capping agent to favour the preferential growth of the particles along one direction, as in the procedure of Mahmoud [14] described in Section 2.2.3, led to the acquisition of much more promising substrates after the deposition on the glass support. An appreciable distribution of the aggregates can be in fact observed in the SEM images of the substrates prepared, for example, via deposition of the colloid concentrated both by a factor of 50 or 5 (Figure 2). The glass surface appears to be rather homogeneously covered by Ag, as shown, for example, in Figure S4 for the substrate obtained via deposition of the colloid concentrated by a factor of 10. Of course, the coating will be more or less dense, depending on the concentration of the deposited colloid.

**Figure 3:**
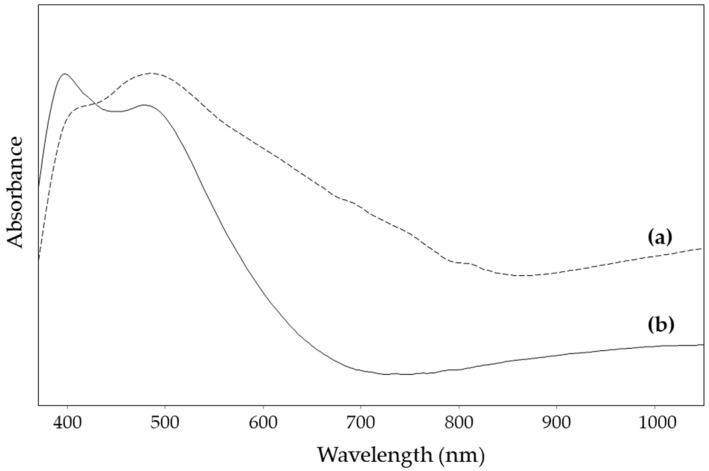
UV–visible reflectance spectra of the AgNR substrate (**a**) concentration factor 1:20 and (**b**) 1:5.

The reflectance visible spectra obtained for the substrates derived from the colloidal suspension concentrated, respectively, 20 or 5 times are shown in Figure 3. For the first concentration, two absorption maxima can be observed, one around 480 nm and the other very broad and centred above 1000 nm. Instead, for the substrate obtained via the deposition of the more concentrated colloid, the first band is broadened on the longer wavelength side and an overall higher absorbance value is obtained in the range of wavelengths examined. This observation suggested the possibility of obtaining a SERS substrate suitable for different excitation wavelengths simply by varying the factor by which this nanorod suspension is concentrated before the deposition, and, on the other hand, the control of the same factor also allowed us, in principle, to obtain an adequate degree of transparency for attempting measurements in the dry state.

The uniformity of the obtained substrates from the point of view of the SERS response was checked by acquiring the spectra of a solution of alizarin from different points of one of the substrates, uniformly distributed on the surface of the deposited drop (Figure S5).

**Figure 4:**
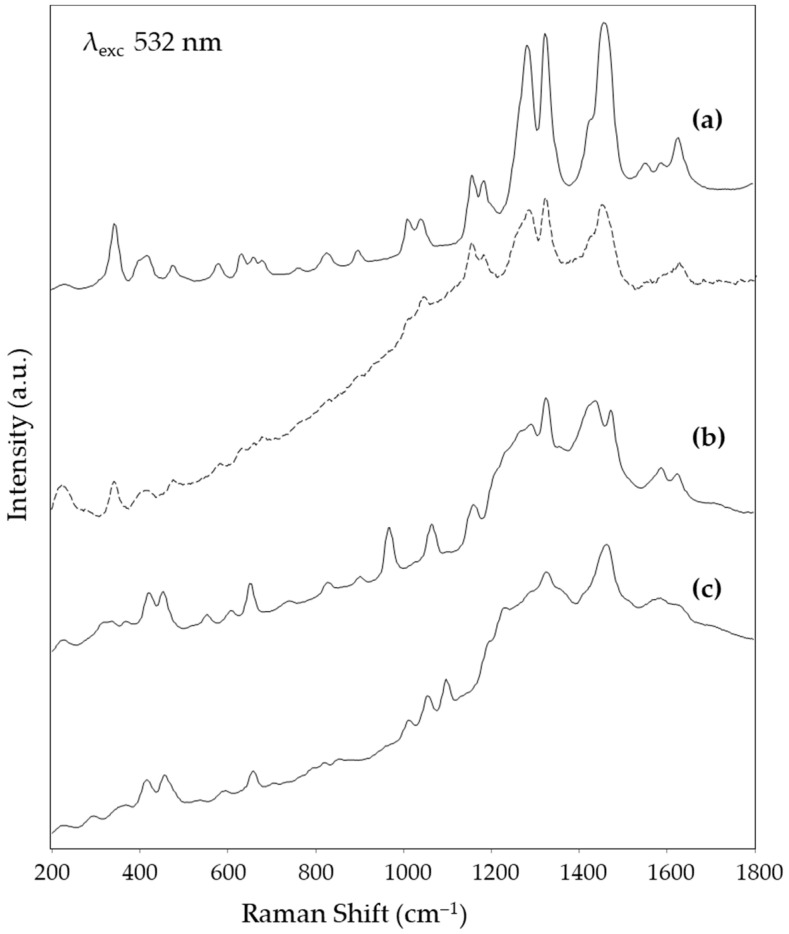
SERS spectra (λ_exc_ 532 nm) obtained from (**a**) alizarin solution (solid line) and in the dry state (dotted line), (**b**) purpurin solution, and (**c**) lac dye solution.

**Figure 5:**
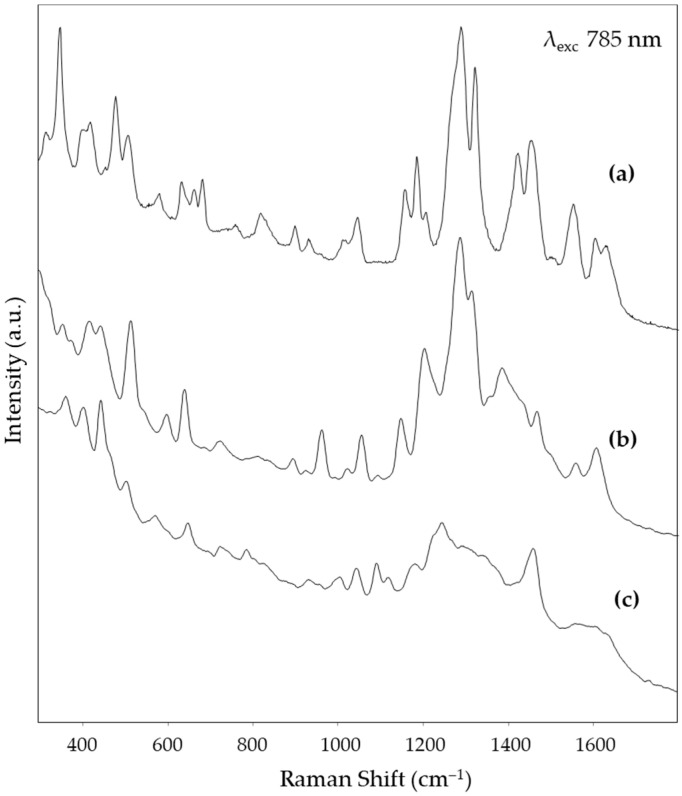
SERS spectra (λ_exc_ 785 nm) obtained from (**a**) alizarin solution, (**b**) purpurin solution, and (**c**) lac dye solution.

**Figure 6:**
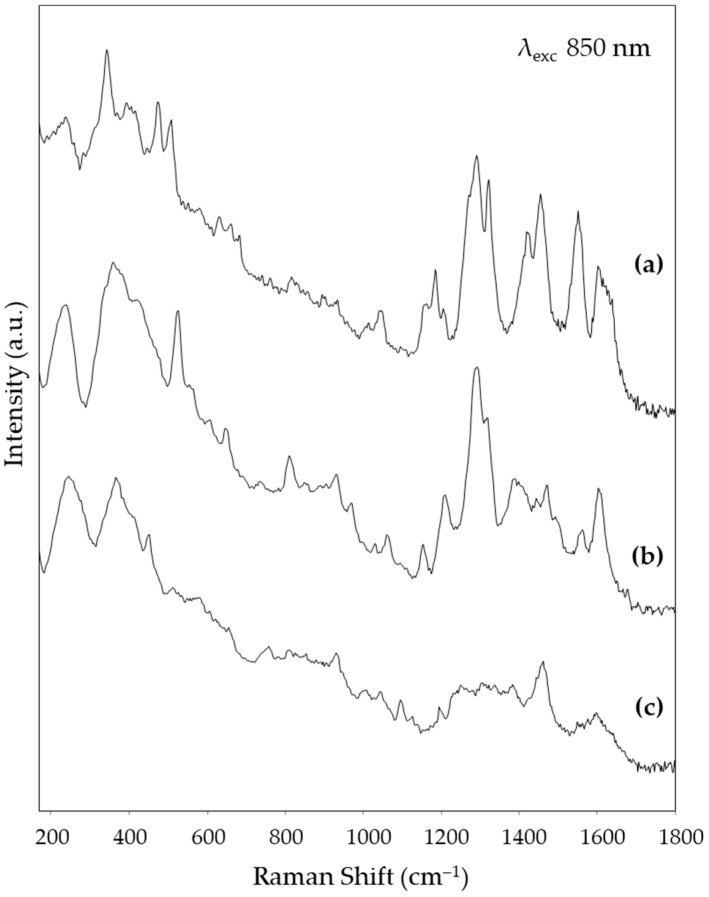
SERS spectra (λ_exc_ 850 nm) obtained from (**a**) alizarin solution, (**b**) purpurin solution, and (**c**) lac dye solution using the SSE™ Raman spectrometer.

Figure 4, Figure 5 and Figure 6 show, respectively, the SERS spectra obtained for the reference solutions of alizarin, purpurin, and lac dye on solid substrates prepared from the Mahmoud nanorod colloid, using the three excitation wavelengths tested in the present work, i.e., 532 nm in the visible region and 785 and 850 nm in the NIR one. The peak wavenumbers are reported in Table 1. For the visible excitation, a substrate derived from the deposition of a colloid concentrated with a factor of 20 was used, while for the longer wavelengths, a concentration factor of 50 gave the best results. It is worth noting that for both excitation at 532 and 785 nm an excellent signal-to-noise (S/N) ratio was obtained, confirming the very good SERS activity of this substrate. In fact, while in the green region of the visible spectrum, the reference dyes examined also exploit the Raman resonance effect, in the NIR region only the enhancement due to the SERS effect is active. A slightly worse S/N ratio is observed for the SERS spectra excited at 850 nm, but the characteristic bands of the three dyes are still well recognizable. The variation in the spectral pattern of each dye, depending on the excitation wavelength, is consistent with the fact that, as discussed above, the resonance effect only contributes to the spectrum excited with green radiation. The observed variations agree with those reported in the literature, respectively, for alizarin [16] and laccaic acid [17].

Finally, the proposed substrate was also shown to be a possible tool for obtaining a SERS response in dry-state conditions. As shown in Figure 4a, a SERS spectrum of the dye from an alizarin-dyed wool thread could be obtained by using the 532 nm excitation and the substrate prepared by depositing the nanorod colloid concentrated with a factor of 1:5. This substrate, in fact, combines the transparency characteristics required by the experimental setup represented in Figure 1 with a plasmonic absorption maximum close to the wavelength of interest. To satisfy this condition at longer wavelengths, a higher concentration factor of the colloid would be required, which would lead to a more covered silver layer. Therefore, it is not suitable for the dry-state experiment. With regards the visible excitation wavelength, however, this substrate adds to that based on Ag nanostars already proposed by some of the authors of the present paper for dry-state SERS analyses [5].

## 4. Conclusions

In the present work, a simple protocol, which does not require complex and expensive methods, has been proposed to prepare SERS-active substrates based on silver nanorods. These nanoparticles, thanks to their anisotropic morphology, are able to interact, forming hot spots without aggregations by electrolytes, unlike the case of Ag nanospheres, thus avoiding inhomogeneity in the final substrates and promoting an intense and uniform SERS response. AgNR were synthesized following three different procedures: two (Rekha and Volkan) specifically designed for SERS applications and involving CTAB as a nanoparticle stabilizer, and one (Mahmoud) presented for a different application, exploiting PVP as a stabilizer and capping agent. The colloids were deposited via drop deposition on a glass slide, functionalized using APTMS, to favour the adhesion of the nanoparticles and their homogeneous distribution over the entire area of the substrate during the drying step. Substrates obtained from Rehka and Volkan AgNR, due to a very low Ag concentration and the presence of residual CTAB, gave a SERS signal for alizarin in methanolic solution with excitation at 532 nm, but neither allow the use of a NIR excitation wavelength or proved to be suitable for dry-state measurements. On the other hand, substrates obtained starting from Mahmoud AgNR performed well in terms of homogeneity and aggregations of the nanoparticles, leading to intense SERS signals from solutions of three reference dyes (alizarin, purpurin, and lac dye) using the excitation at 532 nm. A positive result was also obtained in the dry state, i.e., by analyzing alizarin when absorbed on a woollen thread. Interestingly, by increasing the concentration factor of the deposited nanoparticles, thus favouring their aggregation, it is possible to induce a red shift of the absorption band of these substrates, which makes them also suitable for NIR excitation wavelengths (785 and 850 nm). This property can be useful because, in this way, it is possible to adapt the SERS-active substrates to the Raman instrument available in a given laboratory and facilitate SERS analyses of strongly fluorescent analytes or those inserted in complex and fluorescent matrices. These aspects, combined with the ease of preparation of the proposed AgNR substrate, pave the way for its use for SERS analyses of different compounds in a wide range of application fields.

## Figures and Tables

**Table 1:** SERS bands (cm^−1^) of the dyes—alizarin, purpurin and lac dye in methanolic solution (10^−4^ M)—on solid substrates prepared starting from Mahmoud AgNR.

λ_exc_	532 nm	785 nm	850 nm (SSE™)
Alizarin	343w, 418w, 477w, 581w, 632w, 659w, 680w, 827w, 897w, 1011w, 1042w, 158m, 1186m, 1285s, 1325s, 1459s, 1551w, 1591w, 1629m	346s, 420m, 475s, 507m, 578w, 33m, 662m, 681m, 759w, 817w, 897w, 29w, 1010w, 1049m, 1158s, 183s, 1206m, 1290s, 322s, 1422s, 1458s, 1554s, 603m, 1628m	240w, 343m, 396w, 414w, 476m, 507m, 583w, 633m, 661m, 681m, 817w, 899w, 930w, 1047m, 1158m, 1185m, 1205m, 1290s, 1321s, 1421s, 1453s, 1551s, 1604s, 1627s
Purpurin	318w, 368w, 421m,452m, 554w, 608w, 650m, 827w, 900w, 968m, 1064m, 1161m, 1288s, 1325s, 1435s, 1472s, 1586m, 1624m	312m, 366w, 385w, 427m, 452m, 607w, 649m, 730w, 901w, 968m, 1027w, 1065m, 1099w, 1152m, 1209s, 1294s, 1319s, 1391s, 1470m, 1564w, 1698m	235s, 335w, 357w, 429w, 477w, 524m, 561w, 607w, 649w, 811w, 931w, 969w, 1061w, 1153w, 1209m, 1289s, 1385w, 1445w, 1471w, 1496w, 1560w, 1604m
Lac dye	415w, 455w, 660w, 1013w, 1055w, 1097w, 1325s, 1461s, 1579w	370w, 412w, 453m, 512w, 479w, 658w, 732w, 793w, 938w, 1012w, 1051m, 1099m, 1123w, 1186w, 1249s, 1463s	253s, 366w, 413w, 451m, 512w, 585w, 657w, 759w, 937s, 1008m, 1045m, 1096m, 1195w, 1246m, 1458s, 1605m

Legend: w = weak; m = medium; s = strong.

## Data Availability

Data are available upon the reasonable request from the corresponding author.

## Supplementary Materials

The following supporting information can be downloaded at: https://www.mdpi.com/article/10.3390/nano14221808/s1, Figure S1. UV-visible absorption spectra of (a) Rekha nanorods: silver seeds (pointed line) and nanorods obtained from different amounts of seeds: 625 µL (grey line), 400 µL (black line), and 375 µL (dotted line); (b) Volkan nanorods (dotted line corresponds to the spectrum of seed solution), and (c) Mahmoud nanorods; Figure S2. SEM images of SERS substrates obtained from (a) Volkan AgNR and (b) Rehka AgNR. The EDX spectra of areas rich in CTAB are shown in boxes (c,d) for the two metal films, respectively (the peaks corresponding to bromine are circled in red); Figure S3. SERS spectra (λ_exc_ 532 nm) obtained from alizarin solution on (a) Rekha AgNR (+625 µL seed solution) and (b) Volkan AgNR substrates. Figure S4. (a) SEM image of a part of the substrate obtained by deposition on a glass slide of Mahmoud AgNR concentrated by a factor of 10; (b) X-ray map of Ag; (c) X-ray map of Si. Figure S5. SERS spectra (λ_exc_ 532 nm) obtained from alizarin solution on different micro-areas (approximate diameter 10 µm) uniformly distributed on the surface of the substrate obtained by deposition of Mahmoud AgNR concentrated by a factor of 10. The examined areas are indicated on the figure by coloured dots and the corresponding spectra are shown with the same colour.

## References

[1] Perumal J., Wang Y., Attia A.B.E., Dinish U.S., Olivo M. (2021). Towards a point-of-care SERS sensor for biomedical and agri-food analysis applications: A review of recent advancements. Nanoscale.

[2] Pozzi F., Leona M. (2016). Surface-enhanced Raman spectroscopy in art and archaeology. J. Raman Spectrosc..

[3] Jeanmaire D.L., Van Duyne R.P. (1977). Heterocyclic, aromatic, and aliphatic amines adsorbed on the anodized silver electrode. J. Electroanal. Chem..

[4] Jeon T.Y., Kim D.J., Park S.G., Kim S.H., Kim D.H. (2016). Nanostructured plasmonic substrates for use as SERS sensors. Nano Converg..

[5] Longoni M., Bruni S. (2021). Development of dry-state SERS substrates for the noninvasive detection of artistic dyes in textiles. Opt. Eng..

[6] Garcia-Leis A., Garcia-Ramos J.V., Sanchez-Cortes S. (2013). Silver nanostars with high SERS performance. J. Phys. Chem. C.

[7] Shiohara A., Wang Y., Liz-Marzán L.M., Liz-Marzán L. (2020). Recent approaches toward creation of hot spots for SERS detection. Colloidal Synthesis of Plasmonic Nanometals.

[8] Wang Y., Camargo P.H., Skrabalak S.E., Gu H., Xia Y. (2008). A facile, water-based synthesis of highly branched nanostructures of silver. Langmuir.

[9] Rekha C.R., Nayar V.U., Gopchandran K.G. (2018). Synthesis of highly stable silver nanorods and their application as SERS substrates. J. Sci. Adv. Mater. Devices.

[10] Sancı R., Volkan M. (2009). Surface-enhanced Raman scattering (SERS) studies on silver nanorod substrates. Sens. Actuators B-Chem..

[11] Zhao Y., Kumar A., Yang Y. (2024). Unveiling practical considerations for reliable and standardized SERS measurements: Lessons from a comprehensive review of oblique angle deposition-fabricated silver nanorod array substrates. Chem. Soc. Rev..

[12] Zou S., Ma L., Li J., Liu Y., Zhao D., Zhang Z. (2019). Ag nanorods-based surface-enhanced Raman scattering: Synthesis, quantitative analysis strategies, and applications. Front. Chem..

[13] Gu G.H., Suh J.S. (2010). Silver nanorods used to promote SERS as a quantitative analytical tool. J. Raman Spectrosc..

[14] Mahmoud M.A., El-Sayed M.A., Gao J., Landman U. (2013). High-frequency mechanical stirring initiates anisotropic growth of seeds requisite for synthesis of asymmetric metallic nanoparticles like silver nanorods. Nano Lett..

[15] Israelsen N.D., Hanson C., Vargis E. (2015). Nanoparticle Properties and Synthesis Effects on Surface-Enhanced Raman Scattering Enhancement Factor: An Introduction. Sci. World J..

[16] Lofrumento C., Platania E., Ricci M., Mulana C., Becucci M., Castellucci E.M. (2015). The SERS spectra of alizarin and its ionized species: The contribution of the molecular resonance to the spectral enhancement. J. Mol. Struct..

[17] Cañamares M.V., Leona M. (2007). Surface-enhanced Raman scattering study of the red dye laccaic acid. J. Raman Spectrosc..

