# Supplementary material for: A Tuneable and Easy-to-Prepare SERS Substrate Based on Ag Nanorods: A Versatile Tool for Solution and Dry-State Analyses
Source: Nanomaterials (Basel). 2024 Nov 11;14(22):1808. doi: 10.3390/nano14221808 (PMC11597580; doi:10.3390/nano14221808)
Supplement: Supplementary file 1 [file nanomaterials-14-01808-s001.zip › nanomaterials-3198156-supplementary.pdf]

A tuneable and easy-to-prepare SERS substrate based on Ag-nanorods: a versatile tool for solution and dry-state analyses

Margherita Longoni, Sofia Zucca and Silvia Bruni

Supplementary material

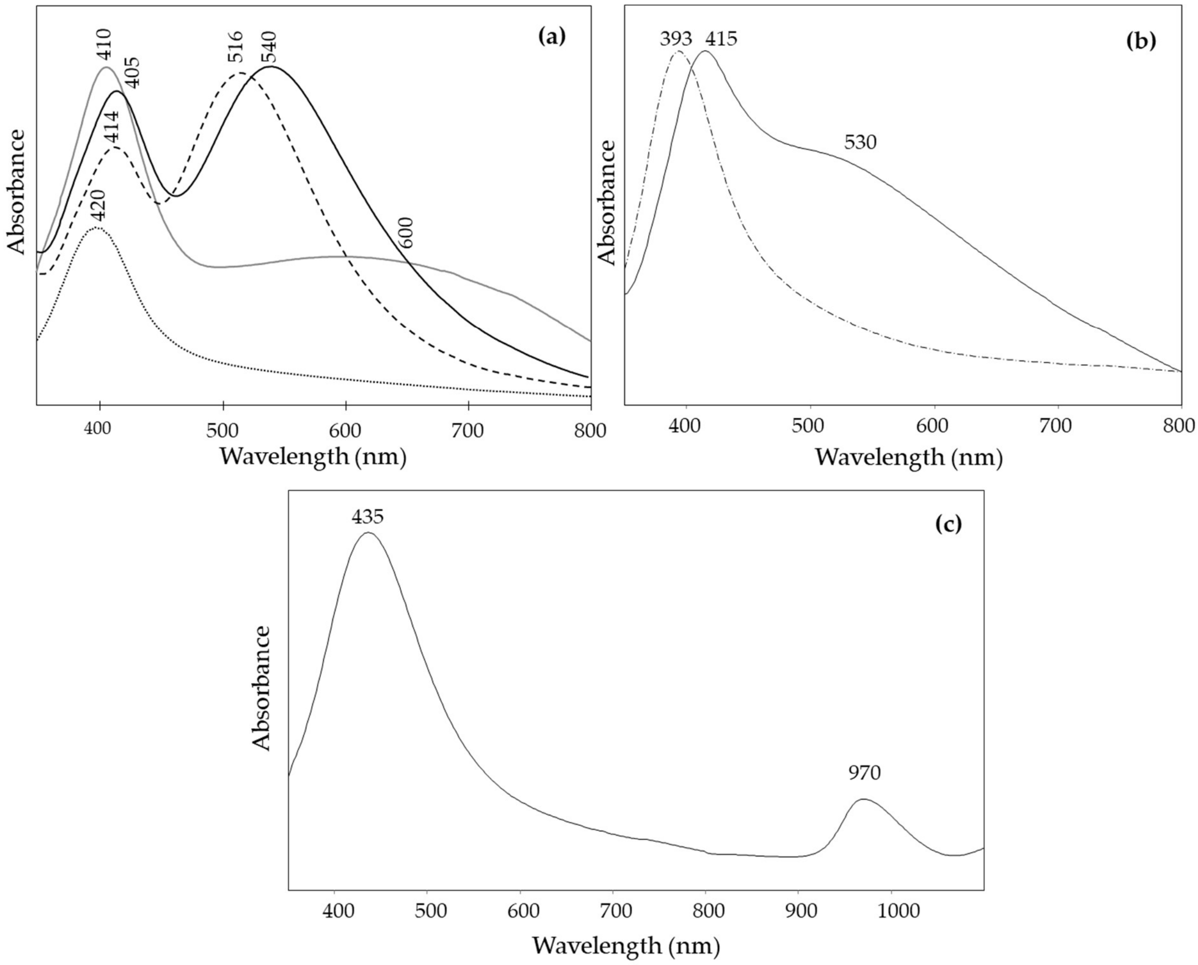

Figure S1. UV-visible absorption spectra of (a) Rekha nanorods: silver seeds (dotted line) and nanorods obtained from different amounts of seeds: 625 μL (grey line), 400 μL (black line) and 375 μL (dashed line), (b) Volkan nanorods (the dashed line corresponds to the spectrum of the seed solution) and (c) Mahmoud nanorods.

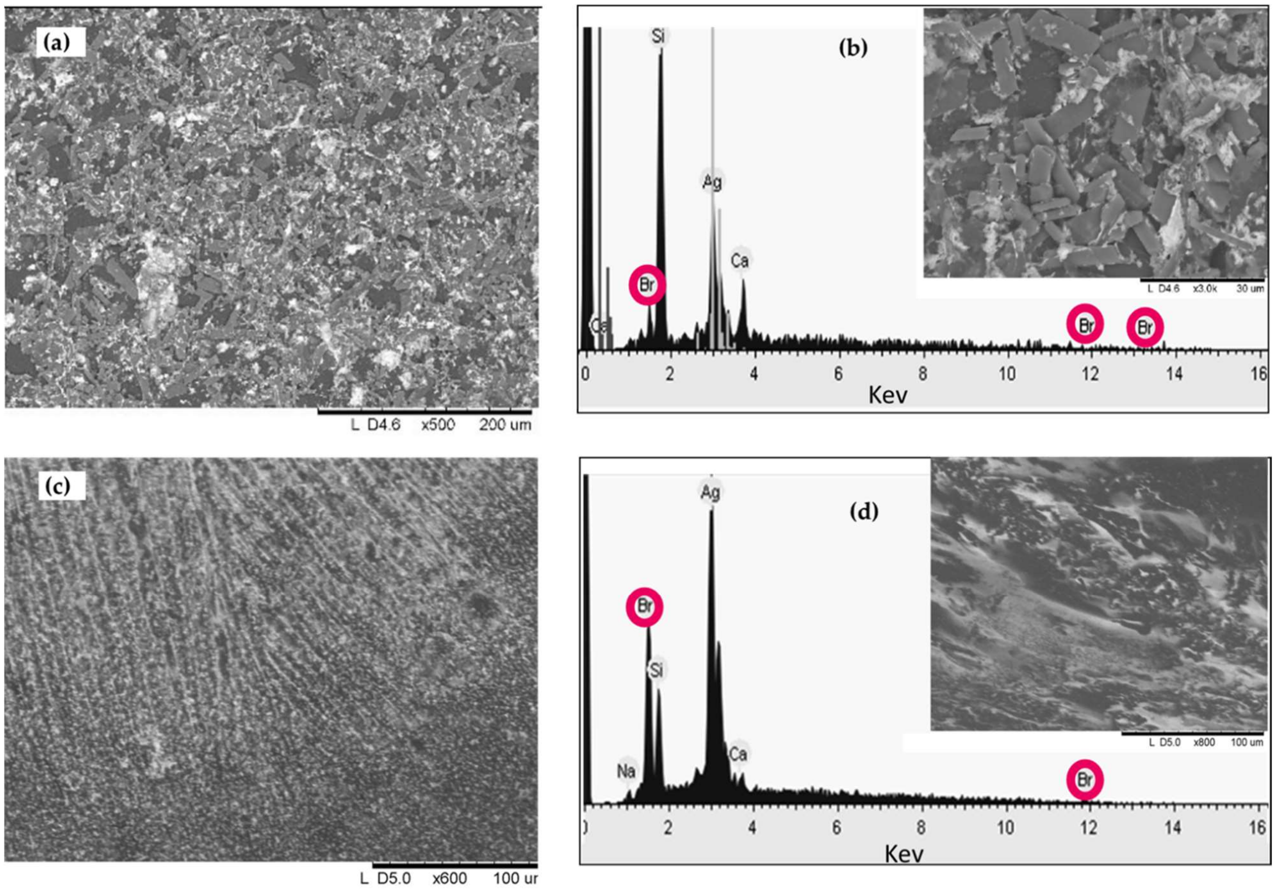

Figure S2. SEM images of SERS substrates obtained from (a) Volkan AgNR (b) Rehka AgNR. EDX spectra of areas rich in CTAB are shown in boxes (c) and (d) for the two metal films respectively (the peaks corresponding to bromine are circled in red).

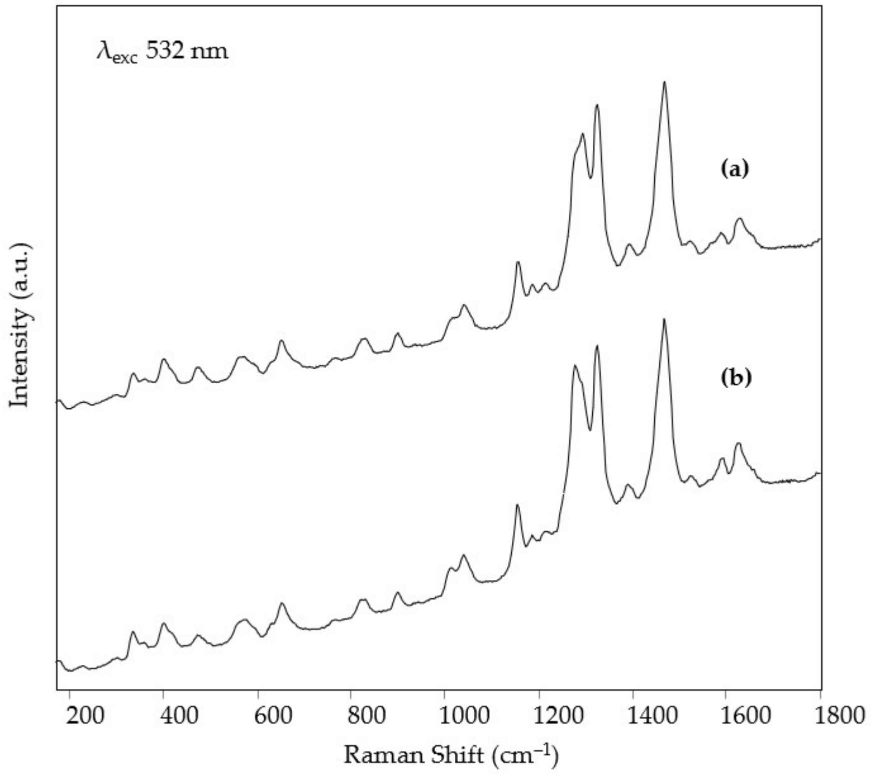

Figure S3. SERS spectra (λ_{exc} 532 nm) obtained from alizarin solution on (a) Rekha AgNR (obtained with 625 μL of the seed solution) and (b) Volkan AgNR substrates.

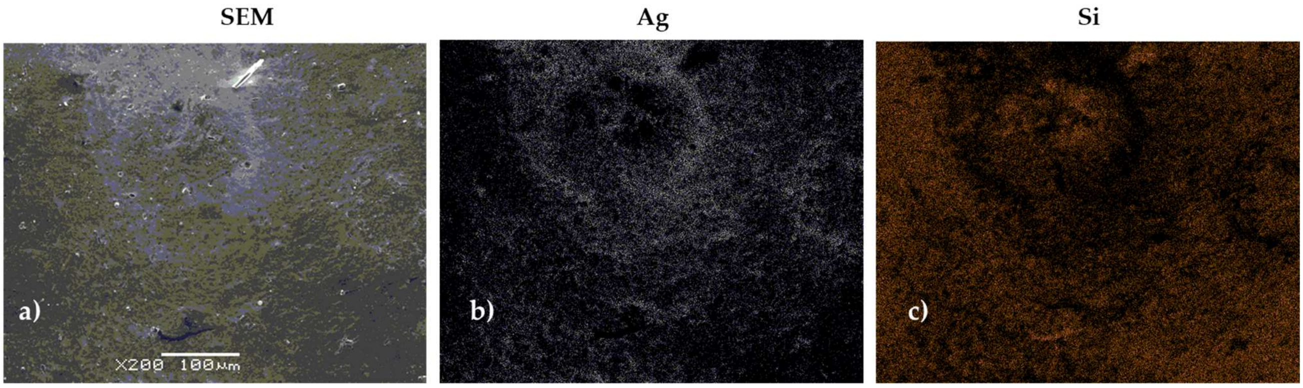

Figure S4. (a) SEM image of a part of the substrate obtained by deposition on a glass slide of Mahmoud AgNR concentrated by a factor of 10; (b) X-ray map of Ag; (c) X-ray map of Si.

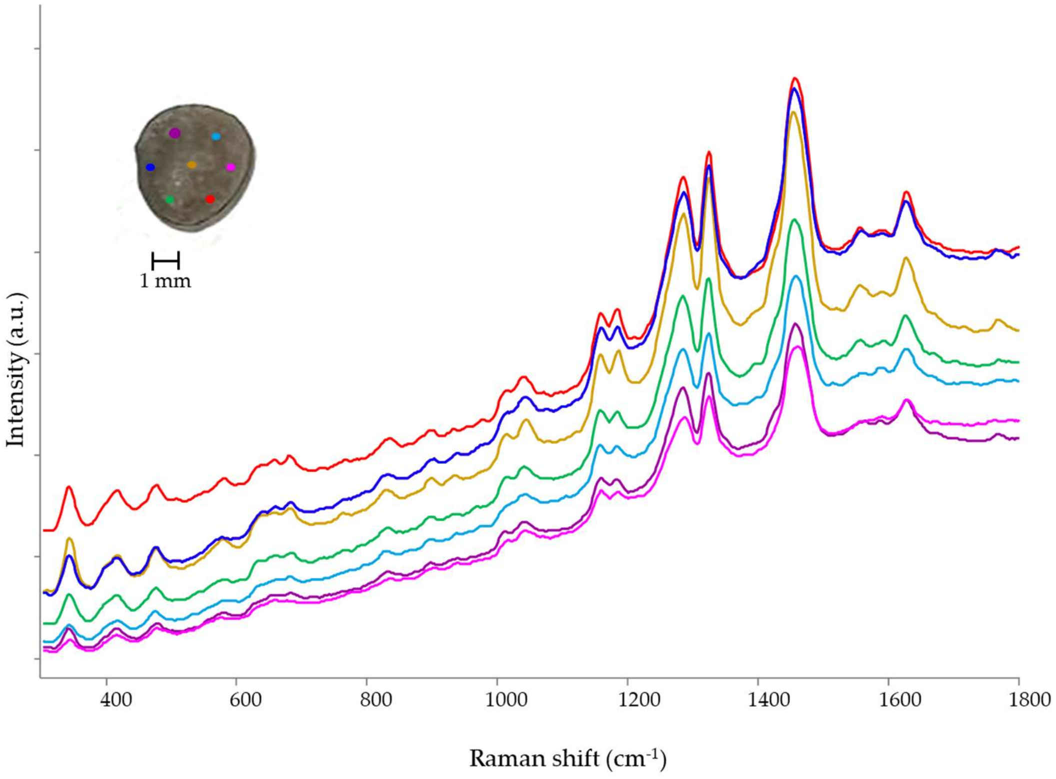

Figure S5. SERS spectra (λ_{exc} 532 nm) obtained from alizarin solution on different micro-areas (approximate diameter 10 μm) uniformly distributed on the surface of the substrate obtained by deposition of Mahmoud AgNR concentrated by a factor of 10. The examined areas are indicated on the figure by colored dots and the corresponding spectra are shown with the same color.
